# Modulation of morphogenesis and metabolism by plant cell biomechanics: from model plants to traditional herbs

**DOI:** 10.1093/hr/uhaf011

**Published:** 2025-01-16

**Authors:** Zhengpeng Wang, Xiaoming Ye, Luqi Huang, Yuan Yuan

**Affiliations:** Experimental Research Center, China Academy of Chinese Medical Science, Beijing 100700, China; State Key Laboratory for Quality Ensurance and Sustainable Use of Dao-di Herbs, China Academy of Chinese Medical Sciences (CACMS), Beijing 100700, China; School of Food and Biological Engineering, Jiangsu University, Zhenjiang, Jiangsu 212013, China; Peking University Health Science Center, Peking University, Beijing 100700, China; State Key Laboratory for Quality Ensurance and Sustainable Use of Dao-di Herbs, China Academy of Chinese Medical Sciences (CACMS), Beijing 100700, China; School of Food and Biological Engineering, Jiangsu University, Zhenjiang, Jiangsu 212013, China; Experimental Research Center, China Academy of Chinese Medical Science, Beijing 100700, China; State Key Laboratory for Quality Ensurance and Sustainable Use of Dao-di Herbs, China Academy of Chinese Medical Sciences (CACMS), Beijing 100700, China

## Abstract

The quality of traditional herbs depends on organ morphogenesis and the accumulation of active pharmaceutical ingredients. While recent research highlights the significance of cell mechanobiology in model plant morphogenesis, our understanding of mechanical signal initiation and transduction in traditional herbs remains incomplete. Recent studies reveal a close correlation between cell wall (CW) biosynthesis and active ingredient production, yet the role of cell mechanics in balancing morphogenesis and secondary metabolism is often overlooked. This review explores how the cell wall, plasma membrane, cytoskeleton, and vacuole collaborate to regulate cell mechanics and respond to mechanical changes. We propose CW biosynthesis as a hub in connecting cell mechanics with secondary metabolism and emphasize that understanding the relationship between mechanical remodeling and secondary metabolism could provide new insights into plant cell mechanobiology and the breeding of high-quality herbs.

## Introduction

Traditional herbs are highly valued for their medicinal and ornamental properties, with their quality determined by organ morphogenesis and the accumulation of active pharmaceutical ingredients [1, 2]. For example, *Panax notoginseng* has characteristic tubercles and their formations directly connect with ginsenosides accumulation [3, 4]; crocetin and crocin determines the specific aroma, flavor, and color of *Crocus sativus* [5]. Recent work on *Salvia miltiorrhiza* has exploited a model in which root phenomics can estimate the production of bioactive metabolites without content determination, providing a direct case of the correlation between organ morphogenesis and secondary metabolism [6]. As research on herb quality spans the organ to cellular levels, more attention should be paid to the linkage between cell population development-induced organogenesis and cell metabolism-mediated bioactive metabolite accumulation at the cellular level, especially from the perspectives of cell wall (CW) development, cell biomechanics, and cell morphology [7, 8]. Studies have shown that CW composition influences both organ morphogenesis and secondary metabolic in herbs [3, 9], suggesting that the CW plays a key role in coordinating organ formation and active ingredient biosynthesis. However, the precise mechanisms by which cell mechanics of plant stem cells regulate cell fate and organogenesis in traditional herbs remain unclear. Moreover, existing research on herbal quality formation has yet to fully integrate the role of cell mechanics, as done in model plants and clinical studies [7, 10].

This review introduces the fundamental aspects of cell mechanics, highlighting its role in modulating morphogenesis and physiological activities. We also discuss how CW biosynthesis links cell mechanics to secondary metabolism and propose strategies for leveraging cell mechanics in molecular breeding to improve agronomic traits and pharmaceutical ingredient production in traditional herbs.

### The key position of cell mechanics in the quality formation of traditional herbs

Traditional herbs generally include specific morphological traits, which represent better quality and make herbs more valuable as a commodity. For example, the scientific connotation of ‘discerning quality by appearance’ in traditional Chinese medicinal herbs is manifested as the organ morphogenesis-mediated ‘excellent shape’ and higher bioactive ingredients-determined ‘high quality’ [2]. Characterizing cell mechanical properties has become an intriguing direction of cell biology; it is focused on the dynamics of biomechanics during cell and tissue developments. Thus, revealing of which will contribute to our understanding of herbal organogenesis.

**Figure 1:**
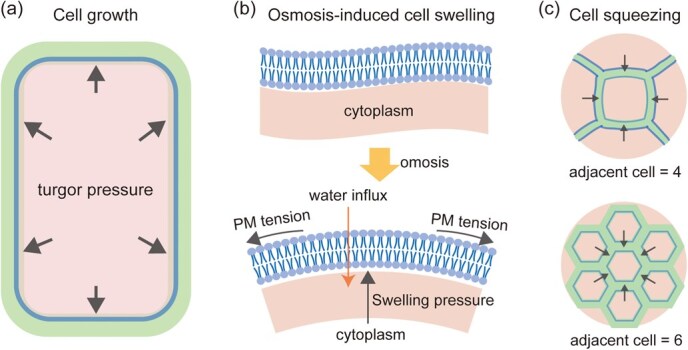
Different mechanical forces on plant cells. (a) Turgor pressure during cell growth. (b) Cell swelling-induced PM tension under hypo-osmosis. (c) Intercellular forces induced by cell squeezing. Black arrows indicate the direction of force.

Plant cell biomechanics are the dynamic forces explaining the resistance and resilience of cell structures under external forces, i.e. the intercellular pressure generated by cell division and mechanical impedance during cell expansion or elongation, and internal forces, i.e. growth-induced turgor pressure and osmosis-induced swelling pressure [11, 12] (Fig. 1). Our current understanding is that plants rely on the CW structure to maintain basic mechanical properties and on the plasma membrane (PM), which contains embedded mechanosensors, to sense mechanical changes and then initiate mechanical remodeling [7, 11, 13]. By monitoring mechanical signals, the plant cell can maintain appropriate geometric and mechanical properties, termed mechanical homeostasis, to meet the requirements of plant growth and development or to adapt to environmental changes [14].

Traditionally, organogenesis determines morphological characteristics and yield of medicinal materials, and secondary metabolism regulates content of active pharmaceutical ingredients, both of which ensures sustainable production of high-quality medicinal materials. Although mechanisms by which cellular mechanical properties regulate plant morphogenesis [7], CW biosynthesis pathways, and cell mechanosensors [13, 15] were well reported in model plants, study on traditional herbs is still lacking. Investigating how mechanical force-induced cell distortion triggers metabolic changes and how these alterations in metabolism reciprocally influence cellular biology has become a hot spot for clinical research on metabolic disorders [10]. In contrast, links between cellular mechanics and secondary metabolism were rarely noted in traditional herbs, which constrains our understanding of through regulating cell mechanics to facilitate the production of active pharmaceutical ingredients and high-quality medicinal materials.

### Factors regulating plant CW mechanics

The CW provides the framework of a plant cell and directly determines the geometrical and mechanical properties of a cell. Due to differences in composition and microfibril arrangement, the primary cell wall (PCW) exhibits relatively higher extensibility to accommodate plasma membrane tension derived from cell turgor pressure, and the secondary cell wall (SCW) confers high rigidity to maintain cell geometry and withstand external mechanical changes [16]. At the subcellular level, CW remodeling confers plasticity and extensibility to CW, which allows cellular deformation activities, such as cell division or cell elongation, to occur [17, 18].

CW remodeling is accompanied by changes in CW mechanical properties, including plasticity, elasticity, and stiffness of CW, which contributes significantly to plant morphogenesis and defense response [12, 19]. Studies on model plants have shown that plant CW mechanics are regulated by CW compounds, structural proteins, and reactive oxygen species (ROS).

#### PCW compounds

The basic compounds of the PCW are cellulose, hemicellulose (xyloglucan, xylan, glucomannan, ß-d-glucan), and pectin, the proportions of which vary depending on the species, tissue, or cell type and contribute to the mechanical properties of CW [17, 20]. During the generation of the plant CW, nascent cellulose molecules assemble into bundles, named cellulose microfibrils, via van der Waals and hydrogen bond interactions [21, 22]. The cellulose microfibrils then interact with other structural polysaccharides to form a 3D grid-like structure, which serves as the skeleton for the PCW [17, 23, 24]. Previous studies on model plants have revealed that the proportions of cellulose, hemicellulose, and pectin substantially affect cell mechanics. For example, CW composition, cellulose microfibrils distribution, and pectin hydration determine distinct flesh viscoelasticity in different groups of apple varieties [25]; the microfibrillar cellulose in the apple parenchyma has more resistance to breaking force after binding to hemicelluloses [26]; for *Arabidopsis* guard cells, the absence of xyloglucan results in a reduction of CW stiffness in *xxt1* and *xxt2* mutants, and the decreased molecular weight of pectin enhances CW elasticity in longitudinal direction in *PGX1-OE* mutants [27].

#### SCW compounds

In the SCW, pectin is largely replaced with lignin, and lignin–polysaccharide interactions have been observed in several studies [28]. The incorporation of lignin into the SCW provides rigidity and hydrophobicity to specific cell types, which contributes to forming apoplast barriers between cells and transporting water in tracheary elements of xylem [29]. The proportion of lignin directly determines the mechanical strength of plants. For example, interfering with lignin synthesis by altering the OsmiRNA166b-*OsHox32* module decreases the mechanical strength of *Oryza sativa* culms [30]. In xylem cells of vascular plants, the hydrophobicity of lignin facilitates water transport, and lignification simultaneously increases the mechanical strength and tolerance to negative hydraulic pressure [31]. Another study revealed that lignin specifically accumulates in abscission zone cells of separated organs during *Arabidopsis* flower abscission, implying that lignified SCW provides transverse shear stiffness for receptacle, which promotes flower shedding [32]. The deposition of lignin is required for the formation of extracellular barriers (e.g. Casparian strip formation or compensatory lignin deposition under absence of Casparian strip [29, 33]) and explosive seed dispersal in *Cardamine hirsute* [34, 35], highlighting the key roles of lignin-associated mechanical properties in plant development.

Suberin and cutin are hydrophobic lipid biopolyesters incorporated into SCW of specialized cell types and tissues, contributing to the formation of hydrophobic CW barriers, which is essential for terrestrial plants to adapt to land environment [36, 37]. Studies have shown that cutin and suberin can deposit on the exterior of epidermal cells to form the cuticle, conferring specific mechanical properties for morphogenesis, according to the root cap cuticle plays a crucial role in lateral root formation [38, 39]. Sporopollenin, a biopolymer composed of polyhydroxylated aliphatic chains and aromatic rings, undergoes rapid deposition during pollen wall development and patterned exine formation [40]. These evidences highlight the contributions of suberin, cutin, and sporopollenin to CW regulation-mediated morphogenesis, but the understanding of their interactions with other CW compounds in modulating CW biomechanics remains insufficient [41].

In brief, the composition is the factor most directly affecting the mechanical properties of the plant CW. The lignified SCW increased the stiffness and rigidity of the plant cell, which enhances the resistance to cell deformation [42], whereas the elasticity and extensibility of the PCW facilitates cell geometry formation and cell growth [43]. Elucidating the mechanical signals that activate lignin biosynthesis will deepen our understanding of how plant cell fate is determined by modulating proportion of the SCW to PCW.

#### CW structural proteins

Various structural proteins also modulate the CW structure, and these proteins apparently influence cell mechanics. Unlike the CW polysaccharides that directly determine mechanical properties of the plant CW, expansins (EXPs) mainly weaken the noncovalent bond between cellulose microfibrils, leading to sliding among structural polysaccharides, which promotes the creeping of the plant CW but has negligible influence on elasticity or plasticity (explanations and differences between creep, extensibility, elasticity, or plasticity of CW have been reviewed by Cosgrove, D. J., [43], Table I) [17, 20]. This expansin-induced CW loosening has no direct association with the tensile stiffness of the CW, but it contributes to CW deformation and remodeling [17, 44, 45]. For example, Samalova *et al*. found that EXPs participates in spatial-specific control of CW viscoelasticity by regulating CW remodeling [46], while Su *et al*. found that the *Solanum lycopersicum* EXP SlExp1 and the cellulase SlCel2 synergistically regulate CW assembly, leading to fruit softening [47]. In addition, α-expansins (EXPAs) regulate CW extensibility via three steps: (1) CW acidification driven by H^+^-ATPases; (2) CW loosening induced by EXPAs; and (3) Sliding of CW structural polysaccharides [13]. Since CW acidification is the fundamental step in EXPAs-induced CW loosening, modulating the pH of CW can affect CW mechanics by altering EXPAs activity [48]. Similarly, ‘acid growth theory’ regarded as CW acidification controls cell elongation via modulating activities of CW structural proteins and CW compounds synthesis enzymes, including EXPs and HG-modifying enzymes [49].

Extensins (EXTs) are hydroxyproline-rich glycoproteins that are responsible for cross-linking the structural polysaccharides on the plant CW and contribute substantially to CW architecture and integrity [50]. There is growing evidence that differences in the hardness of plant tissues are accompanied by differences in the distributions of EXTs, implying that changes in extension-induced CW architecture possibly affect cell firmness [51–53]. Besides EXPs and EXTs, enzymatic proteins associated with CW biosynthesis and deposition, such as β-1,3-glucanase [54], cellulose [55], and pectin methyltransferase [56] affect cellular mechanical properties by changing CW constituents.

#### Reactive oxygen species

ROS homeostasis is of profound significance for the maintenance of CW integrity, biomechanical changes, and synthesis and disassembly in the plant CW, and changes in ROS affect CW mechanical homeostasis. Francoz *et al*. and Dauphin *et al*. reviewed how ROS mediate the cleavage or cross-linking of polysaccharides in the CW microdomain (local differences in CW composition), maintaining the balance between CW loosening and strengthening [18, 57]. ROS also affect callose deposition outside the CW, affecting intercellular signaling and CW mechanics, and contributes to maintaining the activity of the root meristem [58, 59]. In addition, ROS in the apoplast can induce peroxidase-mediated lignin modification during CW remodeling, resulting in biomechanical changes in the plant cell [60–62].

### Synergistic regulation of cellular mechanical properties

Although the CW is the main structure that responds to mechanical changes, PM, cytoskeleton, and vacuole can be coordinated with the CW to regulate the mechanical properties of the plant cell in a more sensitive manner [11].

#### Plasma membrane

Although the PM offers less resistance to compressive stress than the plant CW and therefore makes limited contributions to cellular mechanical strength, the PM is important because it plays an important role in sensing cellular swelling by sensing membrane tension [63]. Previous studies have shown that the plant PM regulates osmotic pressure, turgor pressure, and membrane tension in a subtle balance that drives cell growth [64–67]. Proteins embedded in the plant PM are responsible for sensing signals of cell growth (increased turgor pressure) and environmental changes (osmotic stress or rapid mechanical stimulus) [68, 69]; for example, histidine kinases (e.g. AtHK1) and calcium channels (e.g. OSCA1) sense changes in osmolality [70–72], receptor-like kinases (e.g. RALF34 and THESEUS1) sense defects in the CW integrity [73, 74], and mechanosensitive ion channels (e.g. MSL10) sense membrane mechanics or cell swelling/shrinking [75, 76].

Upon sensing signals that may affect the biomechanical properties of the cell, plant cell initiates the modulation of the PM lipid composition, which alters the mechanical properties of the PM to alleviate membrane tension [69, 77]. In addition, regulation of the membrane voltage (e.g. via ion channels or H^+^-ATPases) maintains the balance between uptake and efflux of cellular solutes, allowing plant cells to respond to osmotic stress or generate the turgor pressure required for cell expansion [64, 78, 79]. The PM maintains close physical contact with the CW and transduces mechanical signals through Hechtian strands (a physical connection between CW and PM during plasmolysis) [80] and surface-anchored proteins (e.g. glycosylphosphatidylinositol-anchored proteins) [81, 82], which enables the CW to directly sense increased turgor pressure or osmotic stress, which in turn drives CW creeping or remodeling in response to cell growth or hyperosmosis-induced plasmolysis.

#### Cytoskeleton

In living plant cells, the cytoskeletal system mainly consists of microtubules and actin microfilaments, which also contribute to the regulation of mechanics-related biological processes in plant cells [83, 84]. Although the cytoskeleton functions in maintaining plant cell geometry, Belteton *et al*. found that the cytoskeleton is not the determinant of cellular shape, but appears to reinforce the mechanical strength of the cell and stabilize geometrical changes because constraining the cell to a specific geometry controls the orientation of the microtubules [85, 86]. However, the cytoskeletal system indirectly regulates cell mechanical properties through CW assembly because cellulose synthase complexes and noncellulosic polysaccharides must be transported by Golgi-derived vesicles along the same direction as the actin microfilament [87, 88]. Different from actin microfilaments, cortical microtubules can determine the orientation of polysaccharides deposited during the development of the nascent CW [89]. In addition, microtubules aligned parallel to the principal direction of anisotropic tensile stress enhance CW resistance to tensile tension [90–92] and determine the assembly orientation of cellulose microfibrils [93, 94], both of which enhance the stability of the cell in the face of acute mechanical stress.

#### Vacuoles

Recent studies have revealed a positive correlation between vacuole swelling and cell expansion, with auxin-induced vacuole volume restriction restricting plant cell size and elongation [95, 96]. The lytic vacuole is present in most types of plant cells, and it has multiple functions, including filling the cytoplasmic space and maintaining ion storage and homeostasis; the vacuole thus regulates turgor pressure and osmotic stress and thereby alters the mechanical properties of the plant cell [11, 97, 98]. The dynamic geometric and quantitative changes of lytic vacuoles (induced by fusion and fission of vacuoles) are specifically present in mechanosensitive cells (e.g. tubular vacuoles and fragmented vacuoles in *Arabidopsis thaliana* root cortical cells), implying that vacuoles participate in sensing cellular mechanics and responding to stimulus in plants [99, 100]. This hypothesis was partially confirmed by Radin *et al*., who showed that mechanical sensing and vacuole tubulation in the pollen tubes of *A. thaliana* require a tonoplast-localized cation channel, PIEZO, and implied that fragmented vacuoles can facilitate mechanical sensing compared to a large central vacuole [101]. Although many proton pumps and ion channels exist in the tonoplast, the vacuole seems to passively maintain mechanical homeostasis of the cytoplasm [102, 103]. Whether vacuoles can spontaneously recognize cell growth signals and initiate an increase in turgor pressure via water uptake should be investigated in further studies.

The CW, PM, cytoskeleton, and vacuole coordinately establish the mechanical integrity of the plant cell. However, our current understanding is mainly based on model plants, and transferring these findings to traditional herbs is challenging. In particular, the immature genetic transformation systems, long-term growth cycles, and specific organ morphologies of medicinal plants increases the difficulty of revealing their mechanobiology. Given the close relationship between cell geometry and turgor pressure, single-cell phenomics offers an effective approach to correlating cell geometry, cell mechanics, and cell metabolism. This was partly utilized in uncovering lignified stone cell formation in pear fruit and the photosynthetic mechanism in microalgae [104, 105]. In addition, the development of a universal computational model may allow easier prediction of mechanical properties of nonmodel plants from their CW metabolic characteristics [8], which would enable the discovery of connections between cell mechanics and organ morphogenesis, growth rate, and the production of active pharmaceutical ingredients.

### Plant cell perception of mechanical signals

To understand how mechanical homeostasis is regulated in plants, it is first necessary to elucidate the mechanisms by which plant cells monitor external and internal mechanical changes, such as growth-induced cell squeezing, expansion-derived turgor pressure, and osmosis-induced cell swelling.

Plant cells perceive mechanical signals in two main ways: via mechanosensitive ion (MS) channels and receptor-like kinases (RLKs) [11]. RLKs and Rapid Alkalinization Factor 4 (RALF4) can sense mechanical changes in the CW by interacting with demethylated pectin; thus, pectin modification connects CW mechanics to mechanosensors [53, 106]. Although RLKs including FERONIA, THESEUS1, and Buddha’s Paper Seal 1 (BUPS1) function as CW mechanosensors, RLKs are both embedded in the PM and connected to the CW polysaccharides and should be regarded as CW–PM proximity sensors [11]. Based on current knowledge, RLKs primarily perceive CW interference (including extracellular mechanical stimulus and breaking of CW integrity induced by abiotic or biotic stress), such as mechanical obstruction of the pollen tube during penetration of the pistil [107], but they may be unable to recognize intracellular mechanical signals, including turgor pressure and osmotic swelling.

MS channels can recognize such signals because cell expansion leads to an increase in membrane tension, which in turn controls the opening of the MS channel. MS channels can perceive forces from the surrounding bilayer through mechanosensitive gating mechanisms. Plant MS channel families are assembled into multiple multimers, including trimers (PIEZO), homotetramer (MCA), pentamers (OSCA), and heptamers (MSL) [108, 109]. Conformational models of two types of MS channels in sensing membrane tension have been elucidated through cryoelectron microscopy: (1) MSL transmembrane domain is assembled as a seven-bladed propeller [108]; (2) OSCA modulate ion permeation via the lipid-plug mechanism [110–112]. It should be noted that plant PIEZO appears localized to the tonoplast rather than plasma membrane [113], and it can modulate vacuole morphology and conduct mechanotransduction during tip growth [75, 101]. When increased membrane tension activates MS channels, ions move along an electrochemical gradient, which then activates a mechanotransduction signaling cascade, allowing plant cells to respond to mechanical alteration. Moreover, MS channels are responsible for responding to continuous mechanical stimuli (e.g. oscillatory motion induced by wind in *Arabidopsis* [114]) and rapid mechanical stimuli (e.g. rapid movements in response to mechanical touch in *Mimosa pudica* [115]).

**Figure 2:**
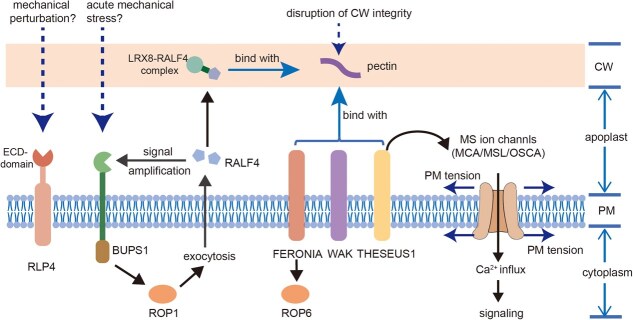
The current understanding of plant mechanosensors on plasma membrane. Abbreviations: BUPS1, Buddha’s Paper Seal 1; CW, cell wall; LRX8, Leucine-Rich Repeat Extensin 8; MCA, Mating Pheromone-Induced Death 1 (MID1)–Complementing Activity; MSL, MscS-Like; OSCA, reduced hyperosmolality induced [Ca^2+^] increase; PM, plasma membrane; RALF4, Rapid Alkalinization Factor 4; RLP4, Receptor-Like Protein 4; ROP, Rho-like GTPase from Plant; WAK, Wall-Associated Kinase.

Here, we summarize representative studies of plant mechanosensors (Table 1) and describe the current understanding of interactions between mechanical changes and mechanosensors (Fig. 2). It is worth noting that it is hard to differentiate CW mechanical (CWM) sensors and CW integrity (CWI) sensors because structural changes in the CW can be regarded as mechanical signal, such as those sensed by RLKs through binding to demethylesterified pectin [106, 116]. This is why current studies use isoxaben treatment, which inhibits CW biosynthesis and deposition, to simulate the mechanical perturbation of CW [117]. However, the isoxaben-induced mechanical changes were hypothesized from changes in CW composition or biosynthesis, and studies directly determining mechanical properties are still lacking. Future studies may need to exploit novel methods to separate mechanical stress from CW integrity to determine if there is direct mechanotransduction between CW and PM, like that mediated by Hechtian strands under plasmolysis.

**Table 1:** Representative mechanosensors in plant cells

**Protein**	**Type**	**Physiological functions**	**Reference**
**Receptor-like kinases (RLKs)**			
THESEUS1	CWI sensor	Linking mechanochemical signaling with cell size for apical hook formation	[116]
FERONIA	CWI/CWM sensor	Essential for sensing CW mechanical perturbations and shaping pavement cells	[106, 117]
BUPS1	CWM/PM mechanic (PMM) sensor	Mechano-transduction during pollen tube penetration of pistils	[107]
**MS ion channels**			
MSL1	PMM sensor	Mechanosensitive opening under increased membrane tension	[108, 118]
MSL10	PMM sensor	Sensing PM tension under mechanical oscillations induced by wind	[114]
		Perception and response to cell swelling under hypo-osmotic shock	[76]
OSCA	PMM sensor	Perception of local membrane deformation caused by force or osmosis	[110]
PIEZO	Tonoplast-localized mechanic sensor	Located in tonoplast; modulates vacuole morphology in caulonemal cells	[101]
		Conduct mechanotransduction in the columella and lateral root cap cells on the root tip	[75]
MCA2	PMM sensor	Activated by membrane tension and voltage	[109]
**Receptor-like protein (RLP)**			
RLP4	CWM sensor	Responds to mechanical changes and controls directional growth	[119]
**Rapid alkalinization factor (RALF)**			
RALF4	CWI sensor	Forms a reticulated pattern with LRX8 and pectin to provide CW mechanical strength during pollen tube growth	[53]
**Wall-associated kinase (WAK)**			
WAK/WAK-like	CWI sensor	Monitor pectin perturbation in the CW	[120, 121]

### The importance of maintaining mechanical homeostasis for plant growth and morphogenesis

Modulating mechanical balance of the plant cell is necessary for adapting to environmental stress or meeting growth and physiological requirements. In this section, we introduce some classical situations in which cell mechanical changes contribute to plant development, morphogenesis, and stress response.

In generally, we hold the opinion that turgor pressure drives cell growth, but the interference of other mechanical forces (e.g. cell squeezing-induced intercellular pressure or penetrative impedance of pistils) makes the situation complicated [12]. Long *et al*. found that a cell in the shoot apical meristem of *A. thaliana* possesses higher turgor pressure when it has fewer neighbors, suggesting that intercellular squeezing pressure hinders turgor-induced cell expansion [122]. Modulation of the specific cell geometry is another way for a plant to relieve mechanical stress due to growth; e.g. puzzle-shaped cells in the epidermis of leaves and cotyledons can limit tensile stress under isotropic growth [123]. In certain circumstances, there may be feedback regulation in response to increasing intercellular pressure. Creff *et al*. reported that the endosperm turgor pressure has two effects: directly driving seed growth and indirectly leading to testa stiffening, which inhibits further seed growth [124].

Changes in mechanical hemostasis also play an essential role in regulating the physiological activities of plants. Synergistic changes of turgor pressure and CW mechanical properties in stomatal guard cells are crucial for stomatal opening and closure [125, 126]. Zhou *et al*. revealed that BUPS1 can sense acute mechanical stress during the *in vivo* penetrative growth of pollen tubes in pistils and subsequently promote CW rigidity to maintain CW integrity [107]. This indicates that the plant cell will spontaneously modulate its own structure to reach mechanical homeostasis.

In some cases, the mechanical structures of a plant CW are adjusted to regulate organ development and morphogenesis. Examples include the protective and promoting effects of the root cap cuticle (an electron-opaque CW modification) during seedling establishment and lateral root emergence [38], the contribution of expansin-β (involved in CW modification and extension) to root architecture modification and nodule formation [127], and the critical role of CW metabolism in modulating fruit softening [128]. The specific organization of cortical microtubules and cellulose microfibrils depends on *KTN1*-regulated CW stiffness, and the mechanical properties of stigmatic papilla cells determine the direction of pollen tube elongation, which indicates that mechanical modulation of the CW can regulate the development of adjacent cells and ultimately regulate the orientation in which plant organs develop [129]. The mechanism underlying the rapid lignification of the endothecium regulates timely anther dehiscence in plant reproduction, suggesting that a rapid alteration in CW composition modulates organ behaviors through significant tissue mechanical variations [130]. Moreover, many mechanical sensors recognize the oscillatory signals in plant cells that, on the one hand, participate in the elongation of tip-growing cells (e.g. Ca^2+^ oscillations promoting pollen tube elongation and root hair growth) [131–133], on the other hand, allows the plant to adapt to oscillatory mechanical stimulations or circadian fluctuations [114, 134].

Most studies of CW mechanics have focused on pollen tube development, fruit ripening, and tip growth because the underlying mechanical changes are easier to define; e.g. the pollen tube suffers mechanical impedance when penetrating the pistil, and the CW softens during fruit ripening. This highlights the importance of selecting appropriate models for evaluating mechanical stress. However, how to measure cell mechanics dynamically and quantitatively in these circumstances still needs to be investigated. Moreover, more complicated physiological processes should also be considered, such as root architecture remodeling and organ primordia formation, because revealing cell biomechanics of traditional herbs aims to breed high-quality herbs with specific organ morphology, better agronomic traits, and higher active pharmaceutical ingredients (e.g., the ideal *Artemisia annua* should have the features of high trichome density, high gr,owth rate, and high artemisinin content [135]). This allows researchers focused on traditional herbs to consider the following questions: (1) How to simulate the specific organogenesis of traditional herbs by geometrical and mechanical properties of plant cells? (2) Do cell geometries and mechanics affect active ingredient production in medicinal organs of traditional herbs? (3) Is it possible to establish a mechanic model to guide oriented cultivation of traditional herbs with high-quality characteristics? Elucidating relationships between cell mechanics-mediated organogenesis and secondary metabolism may give new insights into molecular breeding of high-quality medicinal plants.

### Understanding of domestication through CW development regulation

As the demand for traditional herbs and their active pharmaceutical ingredients increases, there is a growing shift from wild harvesting to large-scale cultivation. This transition requires a deeper understanding of organogenesis to maintain high-quality medicinal materials. Research on domestication of traditional herbs reveals that CW composition connects cell mechanics to secondary metabolite accumulation.

**Figure 3:**
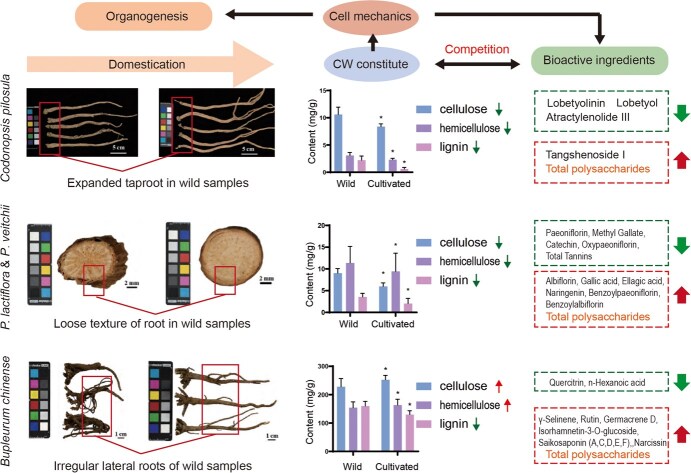
Cell mechanical changes drive variation of morphogenesis and secondary metabolism during the domestication of traditional herbs. (Fig. 3 was redrawn according to figures and data in articles of Lan *et al*., 2024 [136]; Tian *et al*., 2024 [137]; Liu *et al*., 2024 [138]). Abbreviations: *P*. *lactiflora*, *Paeonia lactiflora*; *P. veitchii*, *Paeonia veitchii*.

As shown in Fig. 3, three recent studies on root-herbs including *Bupleurum chinense*, *Paeonia lactiflora*, *Paeonia veitchii*, and *Codonopsis pilosula* reveal that domestication remodels both primary and secondary metabolism by altering CW composition and the content of valuable ingredients, which in turn modifies tissue harness, leading to morphological changes [136–138]. Similar competition between CW development and the accumulation of valuable ingredients was observed in the underground organs of other herbs, such as in the balance between CW development and ginsenoside accumulation during the cultivation of *Panax* species [139, 140]. The growth–defense trade-offs explain these metabolic changes during domestication: alleviation of soil stiffness and microorganism stress remodels the CW mechanics of root-herbs, leading to yield promotion. However, increased CW biosynthesis can hinder the biosynthesis of active pharmaceutical ingredients in traditional herbs [141]. The mechanisms by which traditional herbs remodel tissue mechanics by converting macroscopical soil stiffness into cellular mechanical signals and sensing direction of reduced growth impedance remain unclear.

**Figure 4:**
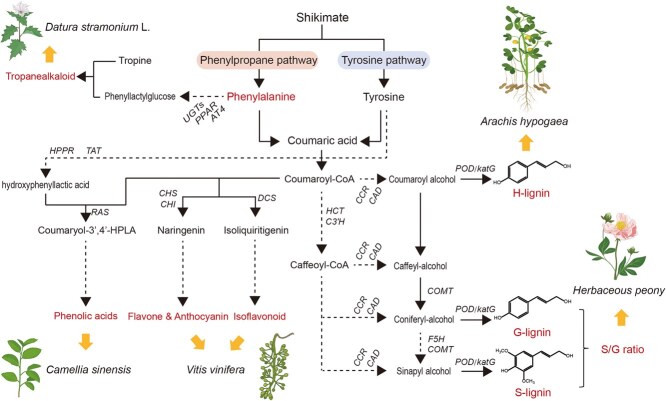
Lignins, flavonoids, phenolic acids, and tropane alkaloids share the phenylalanine pathway in plants. Pathways in Fig. 4 refer to map00940, map00960, map00350, and map00941 in KEGG database. Previous studies revealed the contributions of S/G lignin compositional ratio to stem mechanics of *Herbaceous peony* and H-lignin content to pod size of *Arachis hypogaea* [142–144]. Abbreviations: AT4, aromatic amino acid aminotransferase; CAD, (hydroxy) cinnamyl alcohol dehydrogenase [EC:1.1.1.195]; CCR, cinnamoyl-CoA reductase [EC:1.2.1.44]; CHI, chalcone isomerase [EC:5.5.1.6]; CHS, chalcone synthase [EC:2.3.1.74]; COMT, caffeic acid 3-O-methyltransferase/acetylserotonin O-methyltransferase [EC:2.1.1.68, 2.1.1.4]; C3'H, 5-O-(4-coumaroyl)-D-quinate 3′-monooxygenase [EC:1.14.14.96]; DCS, 6′-deoxychalcone synthase [EC:2.3.1.170]; F5H, ferulate-5-hydroxylase; G-lignin, guaiacyl-lignin; HCT, shikimate O-hydroxycinnamoyltransferase [EC:2.3.1.133]; H-lignin, *p*-hydroxyphenyl-lignin; katG, catalase-peroxidase [EC:1.11.1.21]; HPPR, hydroxyphenylpyruvate reductase, [EC:1.1.1.237]; PAL, phenylalanine ammonia lyase; POD, peroxidase [EC:1.11.1.7]; PPAR, phenylpyruvic acid reductase [EC:4.3.1.24]; RAS, rosmarinic acid synthase; S-lignin, syringyl-lignin; TAT, tyrosine aminotransferase [EC:2.6.1.5]; UGT, phenyllactate UDP-glycosyltransferase.

Another interesting aspect of herbal domestication is the carbon competition between the biosynthesis of CW and valuable ingredients. Polysaccharides are major bioactive compounds in many traditional herbs, including *Astragalus membranaceus*, *Lycium barbarum*, and *Angelica sinensis*, and they share a common monosaccharide metabolic pool with CW polysaccharide biosynthesis [15, 145]. In *S. miltiorrhiza*, different cultivation sites result in different structures of hemicellulose-based polysaccharides, leading to variations in their antitumor activities [146]. Wild *Cochlospermum tinctorium* has higher monosaccharide levels in isolated polysaccharide fractions from its root compared to cultivated samples [147]. These findings suggest that CW biosynthesis acts as a hub linking growth rates, driven by cell mechanics, with the accumulation of active polysaccharides, and adjusting CW biosynthesis could enhance the cultivation of higher quality herbs. For instance, Liu *et al*. found that wild-simulated cultivation of *Dendrobium catenatum* increases the content of nonstarch polysaccharides, resulting in higher quality herbs with better antioxidant activity [148]. Moreover, strengthening the CW with polysaccharides in *O. sativa* improves mechanical strength, biomass production, and salinity tolerance [149]. CW remodeling and sugar accumulation have also been shown to enhance growth and palatability in domesticated citrus fruitlets [150].

Future studies on CW mechanics for high-quality domestication of traditional herbs may focus on: (1) developing methods to quantify the carbon flow competition between CW and secondary metabolite biosynthesis; (2) understanding how plants sense environmental changes through cell mechanical receptors during domestication; and (3) creating mathematical models of cell mechanical dynamics to explain the specific organogenesis of traditional herbs.

### Shared metabolic pathways of CW-associated lignin and phenylalanine-derived metabolites

Lignin is a component of the SCW and largely determines CW mechanical properties, which in turn influence the rigidity of cells and tissues in plants [151–153]. Lignin biosynthesis is closely tied to the biosynthesis pathways of hyoscyamine, flavones, lignans, and phenolic acid because lignin shares the phenylalanine biosynthesis pathway with these phenylalanine-derived secondary metabolites (PDSMs) [154, 155], as shown in Fig. 4. Moreover, lignans are derived from the coupling of phenylpropane units, and coniferyl alcohol serves as a crucial precursor for the biosynthesis of lignan-forming phenylpropane units, S-lignin and G-lignin [156]. Because of this close correlation, it is critical for plants to decide the direction of metabolic flux during different development stages. This process is also of interest to plant biologists because the lignin content and composition determine the lignocellulosic biomass in crops, while high PDSM production determines clinical effect of traditional herbs. Here, we list representative studies investigating how the balance between lignin and valuable PDSMs is regulated (Table 2).

In most circumstances, lignin biosynthesis competes with PDSM production because of the limited amount of total carbon in the plant; thus, regulatory genes in the phenylalanine pathway or transcription factors that regulate both lignin and PDSM biosynthesis genes may not simultaneously promote the biosynthesis of lignin and valuable PDSMs, according to attempts in *O. sativa* or *Malus domestica* [157, 158]. In addition, the subtle carbon flux balance between lignin and valuable PDSMs is subject to dual-direction regulation of transcription factors, according to MYB20, MYB42, MYB43, and MYB85 can positively regulate lignin biosynthesis, while impeding flavonoid accumulation via MYB4-mediated repression of flavonoid biosynthesis in *Arabidopsis* [159]. Interestingly, some PDSMs (e.g. tricin and naringenin) can be incorporated into lignin [157, 160], implying that metabolic competition could potentially be reconciled by shifting monolignol biosynthesis toward PDSM-coupled lignin synthesis, as demonstrated by Hoengenaert *et al*. [161] Moreover, it was reported that different vascular cell types have unique lignin chemistries that confer specific biomechanical and hydraulic properties required for development [162, 163], which demonstrates that investigations on CW mechanic-related PDSM biosynthesis should focus on specific cell types.

### Synergistic promotion of CW biosynthesis and ginsenoside production

Ginsenosides are specifically produced by *Panax* plants and are well known for their antifatigue and antineoplastic activities. Previous studies showed that ginsenosides are defensive compounds in *Panax* spp., but they are also autotoxic compounds because ginsenoside can lead to ROS accumulation and degradation of the CW of both plants and invasive pathogens[164, 165] . However, how *Panax* plants balance CW remodeling and ginsenoside production to balance growth and defense is still unclear. It has been revealed that overexpression of *PqEXLA1* and *PqEXLB1* (EXP-like subfamily genes) in *Panax quinquefolius* can increase CW thickness and cell volume but reduce biosynthesis of the ginsenoside Rg1, which implies competition between CW remodeling and Rg1 accumulation [140]. Similarly, deletion of genes encoding enzymes acting on UDP-glucose as a substrate (*FKS1*, cell wall biosynthesis; *GLC3*, glycogen biosynthesis; *ALG5*, protein glycosylation) to increase the production of ginsenoside compound K in engineered yeast cells revealed competition between CW biosynthesis and ginsenoside biosynthesis because the UDP-glucose pool connects the two biosynthetic directions [166].

**Table 2:** Recent studies of the correlation between lignin and PDSM biosynthesis

**Species**	**Material**	**Lignin changes**	**PDSM changes**	**Reference**
*Oryza sativa*	*OsCHS1*-deficient mutant (T-DNA insertion)	Decreased lignin-integrated tricin signals	Decrease in apigenin, luteolin, chrysoeriol, isovitexin, orientin, and isoorientin;	[157]
	*OsCHI*-deficient mutant (CRISPR/Cas9)	Lower contents of CW lignin and thioacidolysis-derived lignin monomers	Decrease in tricin (CW-bound flavonoids) content	[157]
	*OsCHIL*-deficient mutant (CRISPR/Cas9)	Higher contents of CW lignin and thioacidolysis-derived lignin monomers; lower S/G ratio	Decrease in apigenin, luteolin, chrysoeriol, isovitexin, orientin, isoorientin contents;	[157]
*Scutellaria baicalensis*	Kuqin (rotten xylem) vs Ziqin (strip-type)	Decrease in lignin biosynthesis	Increase in wogonin, baicalin, and eriodictyol contents; Decrease in baicalein and apigenin	[9]
*Chrysanthemum morifolium*	*CmMYB8*-overexpression mutant	Decrease in lignin content and S/G ratio	Decrease in rutin, isorhamnetin, quercetin, and kaempferol	[167]
*Malus domestica (‘Stolav’ apple)*	*MdMYB16*-RNAi, *miR7125-*overexpression and *MdCCR*-RNAi apple fruits	Decrease in lignin content	Promotion of anthocyanin accumulation and fruit coloration	[158]
	MdMYB16-overexpression, miR7125-RNAi, and MdCCR-overexpression apple fruits	Increase in lignin content	Reduced anthocyanin accumulation	[158]
*Punica granatum*	*PgMYB308-like*-overexpression in pomegranate hairy roots	70% increase in total lignin content; 4.8-fold increase in shikimate	Reduced contents of several flavonoids and hydrolyzable tannins	[168]
*Populus alba × grandidentata*	*MdCHS3*-poplar (*MdCHS3* derived from *Malus domestica*)	Reduced acid-insoluble lignin; increased alpha cellulose	Promotion of naringenin production	[160]
*Fritillaria unibracteata*	*FuPAL1* transgenic *A. thaliana*	Higher lignin content in vascular bundles, xylem, and cortex	Fewer total flavonoids;	[169]
*Angelica sinensis*	*A. sinensis* seedlings at the bolting stage	Increase in lignin accumulation	Reduced ferulic acid and flavonoid accumulation	[170]
*Arabidopsis thaliana*	*MYB20/42/43/85* quadruple mutant (T-DNA insertion)	Reduction in lignin	Promotion of anthocyanin and soluble flavonoid accumulation	[159]
*A. thaliana*	*F6′H1_COSY*-overexpression mutant	Reduction in Klason lignin, increase in S/G ratio	Promotion of scopoletin and scopolin production	[161]
*Artemisia annua*	AaCAD-overexpressing *A. annua*	Increased lignin content in stems and branches	Increase in coumarin; decrease in artemisinin and arteannuin B	[171]

**Figure 5:**
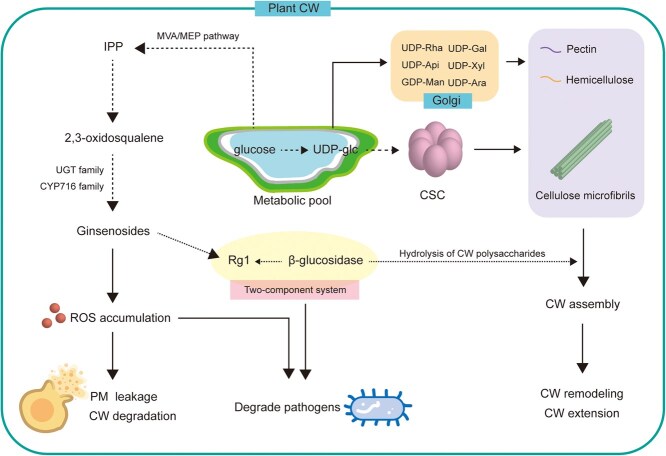
A metabolic pool connects cell wall biosynthesis and ginsenoside biosynthesis in *Panax* spp. Abbreviations: CW, cell wall; PM, plasma membrane; UDP, uridine diphosphate; UDP-Glc, UDP-D-glucose; UDP-Rha, UDP-L-rhamnose; UDP-Api, UDP-D-apiose; UDP-Xyl, UDP-D-xylose; GDP-Man, GDP-D-mannose; UDP-Ara, UDP-L-arabinose; CSC, cellulose synthesizing complex (typically consists of a heterotrimeric cellulose synthase [EC2.4.1.12] core); IPP, isopentenyl pyrophosphate; ROS, reactive oxygen species; UGT, UDP-glucosyltransferase.

Although competitive biosynthesis poses a challenge to simultaneously promoting cell growth and ginsenoside accumulation (Fig. 5), two recent studies revealed that specific transcription factors have the potential to solve this problem. Jiao *et al*. discovered that PgMADS41 and PgMADS44 can change the adventitious root morphology of *Panax ginseng* by activating the expression of *PgEXLB5* and *PgEXPA13*, and promote production of the ginsenoside Ro by upregulating *SE4*, *β-AS13*, and *CYP716A52v2* [172]. Yu & Hua reported that PnPHL8 modulates the CW architecture and CW constituents (cellulose and callose) by upregulating *PnGAP* and *PnEXPA4*; meanwhile, it increases the content of the ginsenoside Rb1 by upregulating *GGPPS* and *CYP716A47* in *Panax notoginseng* [139].

Some CW-related enzymes can act on the ginsenoside biosynthesis pathway in *Panax* plants. Overexpression of *PAL* (2.63-fold higher expression compared with control root culture), which is involved in SCW biosynthesis and xylem cell expansion, upregulates the expression of *Pq3-O-UGT2* (3.73-fold higher) and increases the content of the ginsenoside Rg3 (6.19-fold higher) in transgenic *P. ginseng* root culture [173], suggesting that key SCW biosynthesis genes can also modulate the direction of metabolic flux to ginsenoside synthesis. In addition, a novel two-component system responsible for chemical defense in *Panax* spp. associates CW biosynthesis with ginsenoside metabolism. The selective hydrolysis of 20(*S*)-protopanaxadiol ginsenosides catalyzed by PnGH1 (a β-glucosidase that generally participates in trimming the xyloglucan backbone [174] and synthesizing the intine CW [175]) contributes to the production of less polar ginsenosides with higher antifungal activity, suggesting the potential dual functions of β-glucosidase in CW and ginsenoside metabolism [176].

Although the aforementioned works demonstrated the subtle mechanism underlying the regulation of CW and ginsenoside biosynthesis in *Panax* spp., no studies have directly evaluated how cell mechanical properties change during CW remodeling or determined whether modulating the CW mechanics-associated texture of *Panax* roots (e.g. grain and rigidity) determines the accumulation or distribution of ginsenosides in *Panax* plants. Identifying dual-function genes will help us understand how to direct the metabolic flux toward CW and ginsenoside biosynthesis, thus accelerating germplasm improvement and contributing to intelligent production of ginsenosides in *Panax* plants.

## Concluding remarks and future perspectives

Recent exciting recoveries have revealed how the unique mechanical properties of plant cells are established. Compounds, structural proteins, and ROS play important roles in CW remodeling, which regulates the fundamental mechanics of a plant cell. At the same time, the CW combined with the PM, cytoskeleton, and vacuole enables precise sensing and feedback of mechanical changes. However, much remains to be deciphered in the near future. For example, different mechanosensor-induced signaling cascades need to be more deeply compared. Resolving the initiation, sensing, and transduction mechanisms for cell mechanical signals would constitute a major breakthrough for cell developmental biology. There are also some methodological problems that need to be solved. For example, a real-time method to record changes of cell mechanical properties is still lacking. In addition, current knowledge about cell mechanics is based on a few model plants; can we integrate previous experimental data to construct a universal model (maybe with the assistance of artificial intelligence) for more easily predicting cell mechanical properties, like Zhang *et al*. did for the *Arabidopsis* CW? [8] Establishing these methods will greatly accelerate research on cell mechanobiology.

Current understanding of cell mechanobiology in model plants paves the way for revealing organ morphogenesis and mechanical signal transduction mechanism of traditional herbs. Oriented cultivation of medicinal organs and organoids with specialized morphology is a promising research direction. Exploration of CW biosynthesis in model plants will deepen our understanding of traditional herbs, which may facilitate effectively producing herbal resources through CW-mediated growth and defense balance. However, the complex biosynthesis pathway of active pharmaceutical ingredients, specific organ morphologies, and unique biological function of bioactive ingredients in regulating plant development cannot be directly validated in model plants. Transplant methodologies used in model plants can facilitate investigation of cell mechanical properties. However, the methodology for elucidating relationships between cell mechanics-mediated organogenesis and secondary metabolite accumulation requires targeted innovation.

For breeding of traditional herbs, although we outlined the competition for carbon between CW biosynthesis and biosynthesis of active pharmaceutical ingredients, what is intriguing to us is how to balance the flux between these pathways because CW synthesis-mediated cell development and accumulation of secondary metabolites are both important for the production of pharmaceutical ingredients. Discovering transcription factors and functional genes redirecting metabolic flux to balance these two processes will greatly contribute to molecular breeding of medicinal plants. In addition, the morphogenesis of different organs induced by changes in cell geometry and mechanical properties possibly modulate the accumulation and distribution of active pharmaceutical ingredients, but there is currently no direct evidence for this. Moreover, several studies have reported that active pharmaceutical ingredients participate in plant development and defense, and whether active pharmaceutical ingredients participate in remodeling cell mechanics will be an interesting question to explore.

In conclusion, the basic framework of plant cell mechanobiology has been established in the past decades. Future work should be aimed at understanding the intertwinement of cell mechanics, organ morphogenesis, and plant metabolism from an integrated perspective.
